# Cardiac MRI underlines the role of BNP and hematologic parameters as heart failure markers in patients with Ebstein's anomaly of the tricuspid valve

**DOI:** 10.1186/1532-429X-16-S1-O107

**Published:** 2014-01-16

**Authors:** Michael Steinmetz, Olga Becker, Thuy T Nguyen, Peter Lauerer, Martin Fasshauer, Christina Unterberg-Buchwald, Andreas Schuster, Wieland Staab, Joachim Lotz, Thomas Paul, Jan M Sohns

**Affiliations:** 1 Pediatric Cardiology and Intensive Care, Georg-August-University, Goettingen, Lower-Saxony, Germany; 2 Cardiology and Pneumology, Gerorg-August-University, Goettingen, Germany; 3 Diagnostic and Interventional Radiology, Georg-August-University, Goettingen, Lower-Saxony, Germany; 4 partner site Göttingen, DZHK (German Cardiovascular Research Center), Göttingen, Germany

## Background

Ebstein's anomaly of the tricuspid valve involves a congenitally displaced and dysplastic tricuspid valve (TV). The displacement of the TV towards the apex results in an enlarged right atrium (RA) with an atrialzied portion of the right ventricle (aRV) and an enlarged functional right ventricle (fRV). Brain natriuretic peptide (BNP) is a heart failure marker. Its role in Ebstein's anomaly has not been evaluated in conjunction with cardiac MRI (CMR) parameters. Hematologic parameters (hemoglobin (Hb), hematocrite (Hct)) are upregulated in states of pulmonary hypo perfusion as in congenital cyanotic heart disease. Intermittent Pulmonary hypo perfusion may be a an indicator of right heart failure in patients with Ebstein's anomaly, but has not been studied so far. The aim of the present study was to correlate CMR functional parameters and severity of disease with BNP and hematologic parameters in patients with Ebstein's anomaly.

## Methods

26 patients with non-corrected Ebstein's anomaly were studied prospectively. Laboratory parameters (BNP, Hb, Hct), CMR data (RA, aRV, fRV, left atrial (LA) and left ventricular (LV) volumes and functional parameters like ejection fraction (EF), stroke volume (SV) and others) as well as exercise capacity (maximal oxygen uptake (VO2 max.), CO2 equivalent (VE/VCO2), maximum heart rate (max. HR) using a cycle ergometer) were all measured on the same day for an individual patient.

## Results

Mean patient age was 25+-14 years (range: 10 - 60). fRV enddiastolic volume index (EDVi) was increased to 118.3+-57.0 ml/m2 (ref. value RV EDVi 67-111 ml/m2). Plasma BNP-level was increased (mean: 74.3+- 123.1 ng/l), in 15% of patients markedly above the heart failure level. Hb and Hct were increased above normal levels in 20% and 23% of patients, respectively. VO2 max. was decreased to 67.1+- 22.1%, VE/VCO2 increased to 117.8+-36.1%, both of predicted normal values. Transcutaneous SaO2 was normal (98+-2.8%). Statistical analysis revealed a positive correlation of BNP and RA EDVi (p = 0.00001), fRV EDVi (p = 0.00001) and VE/VCO2 (p = 0.022), a negative correlation with LV EF (p = 0.0003) and max. HR from exercise testing (p = 0.002). Similar correlations were found for Hb and Hct. Multiple regression analysis identified BNP as an independent predictor of RA EDVi, fRV EDVi, LV EF from CMR, as well as max. HR from exercise testing. Hkt and Hb were independent predictors of fRV EF and RA SV. The higher either BNP levels, Hct or Hb were in our study population, the lower were cardiac function on CMR and physical exercise capacity during exercise testing.

## Conclusions

To the best of our knowledge, this is the first prospective study that shows a correlation of functional data from CMR and exercise testing with BNP levels and hematologic parameters -as a measure of pulmonary perfusion- in patients with Ebstein's anomaly. BNP and the studied hematologic parameters (Hb, Hct) may be useful prognostic markers in patients with Ebstein's anomaly.

## Funding

None to disclose.

